# Complete Sequencing of pNDM-HK Encoding NDM-1 Carbapenemase from a Multidrug-Resistant *Escherichia coli* Strain Isolated in Hong Kong

**DOI:** 10.1371/journal.pone.0017989

**Published:** 2011-03-21

**Authors:** Pak Leung Ho, Wai U. Lo, Man Kiu Yeung, Chi Ho Lin, Kin Hung Chow, Irene Ang, Amy Hin Yan Tong, Jessie Yun-Juan Bao, Si Lok, Janice Yee Chi Lo

**Affiliations:** 1 Department of Microbiology, The University of Hong Kong, Hong Kong, Special Administrative Region, People's Republic of China; 2 Carol Yu Center for Infection, The University of Hong Kong, Hong Kong, Special Administrative Region, People's Republic of China; 3 Genome Research Centre, The University of Hong Kong, Hong Kong, Special Administrative Region, People's Republic of China; 4 Public Health Laboratory Services Branch and Department of Health, Centre for Health Protection, Hong Kong, Special Administrative Region, People's Republic of China; Hopital Raymond Poincare - Universite Versailles St. Quentin, France

## Abstract

**Background:**

The emergence of plasmid-mediated carbapenemases, such as NDM-1 in *Enterobacteriaceae* is a major public health issue. Since they mediate resistance to virtually all β-lactam antibiotics and there is often co-resistance to other antibiotic classes, the therapeutic options for infections caused by these organisms are very limited.

**Methodology:**

We characterized the first NDM-1 producing *E. coli* isolate recovered in Hong Kong. The plasmid encoding the metallo-β-lactamase gene was sequenced.

**Principal Findings:**

The plasmid, pNDM-HK readily transferred to *E. coli* J53 at high frequencies. It belongs to the broad host range IncL/M incompatibility group and is 88803 bp in size. Sequence alignment showed that pNDM-HK has a 55 kb backbone which shared 97% homology with pEL60 originating from the plant pathogen, *Erwina amylovora* in Lebanon and a 28.9 kb variable region. The plasmid backbone includes the *mucAB* genes mediating ultraviolet light resistance. The 28.9 kb region has a composite transposon-like structure which includes intact or truncated genes associated with resistance to β-lactams (*bla*_TEM-1_, *bla*_NDM-1_, Δ*bla*_DHA-1_), aminoglycosides (*aacC2*, *armA*), sulphonamides (*sul*1) and macrolides (*mel*, *mph2*). It also harbors the following mobile elements: IS*26*, IS*CR1*, *tnpU*, *tnpAcp2*, *tnpD*, Δ*tnpA*Tn*1* and *insL*. Certain blocks within the 28.9 kb variable region had homology with the corresponding sequences in the widely disseminated plasmids, pCTX-M3, pMUR050 and pKP048 originating from bacteria in Poland in 1996, in Spain in 2002 and in China in 2006, respectively.

**Significance:**

The genetic support of NDM-1 gene suggests that it has evolved through complex pathways. The association with broad host range plasmid and multiple mobile genetic elements explain its observed horizontal mobility in multiple bacterial taxa.

## Introduction

The development of carbapenem resistance among *Enterobacteriaceae* is a major public health threat because carbapenems are currently the cornerstone therapy for patients with serious and life-threatening infections caused by strains producing extended-spectrum β-lactamases (ESBLs). In the last decade, the ESBL-producing organisms, especially those carrying the CTX-M group of β-lactamases rapidly increased and became endemic in many countries. In Asian countries, high rates of CTX-M positivity have been found among both hospital and community isolates [1]. In Hong Kong, over 80% of the ESBL-producing *Enterobacteriaceae* clinical and faecal isolates were found to be CTX-M positive [2]–[4]. CTX-M-14 was the major type although a range of other alleles (CTX-M-1, 3, 13, 15, 24, 27, 38, 55, 57 and 64) have also been reported [2]–[4]. Sporadic isolates of *Enterobacteriaceae* have been found to have plasmid-mediate carbapenemases but they remained rare [5]. Consequently, many public health authorities have expressed serious concerns when an international group of researchers reported the findings for patients with infections by carbapenem-resistant *Enterobacteriaceae* (CRE) carrying the novel resistant gene, NDM-1 (for New Delhi metallo-β-lactamase) with a putative link to the Indian subcontinent, where the burden of CTX-M producing bacteria is substantial [6]. The authors identified 44 isolates with NDM-1 in Chennai, 26 in Haryana, 37 in the UK, and 73 in other sites in India and Pakistan. The 37 isolates from UK were from 29 patients of which 17 (59%) had travelled to India or Pakistan within the past year [6]. In another study of 39 CRE isolates recovered during 2006–2007 in India, 15 strains were found to carry NDM-1 and 10 additional harbored OXA-181 [7]. NDM-1 was first reported in Sweden in a *Klebsiella pneumoniae* strain (05-506) derived from the urine culture of a patient of Indian origin in January 2008 [8]. The same gene was carried by a *E. coli* strain in the gut of the same patient. However, subsequent work showed that NDM-1 probably emerged earlier before its first recognition in Sweden in 2008 [9]. Other countries and areas which have detected NDM-1 included the United States, Canada, Australia, Germany, Japan and Hong Kong [10]. The horizontal mobility of the NDM-1 gene involving different plasmids play a major role in the dissemination of this resistance determinant but clonal spread and involvement of the globally disseminated ST258 *Klebsiella pneumoniae* lineage has also been reported [9], [11].

NDM-1 was originally found on a plasmid with size of ca. 180 kb but the incompatibility group (Inc) could not be defined [8]. In the UK and Indian Subcontinent, NDM-1 was found on plasmids of various sizes (ca. 50 to 300 kb) which belonged to at least three different Inc groups including A/C, FI/FII and an undefined type [6]. In Hong Kong, a NDM-1 positive *E. coli* strain was isolated in October 2009. It was identified by retrospective testing of an *E. coli* isolate (HK-01) with reduced susceptibility to imipenem [5]. Here, we reported the complete sequencing of the plasmid carrying the NDM-1 gene in the isolate.

## Results

### Conjugation

Conjugation experiments were conducted using different antibiotics in combination with sodium azide for selection of transconjugants. In general, frequencies of transfer for filter and solid mating were similar. The transfer frequencies obtained by using meropenem were variable, ranging from <10^−8^ to 10^−3^ per donor cell. Conjugation was more likely to be successful at lower meropenem concentration (<0.5 µg/ml) than when higher concentration (1 and 2 µg/ml) was used for transconjugant selection. However, high transfer frequencies were consistently obtained when other antibiotics, ampicillin, gentamicin and amikacin were used. In all the transconjugants, a single plasmid of about 90 kb was transferred. All transconjugants had reduced susceptibility or resistance to the carbapenems (imipenem, meropenem, ertapenem, doripenem) relative to the recipient strain. For the non-β-lactam antibiotics, an identical resistance profile was shared by all the transconjugants, being resistant to gentamicin, netilmicin, tobramycin and amikacin but susceptible to ciprofloxacin, tetracycline, sulphonamides, trimethoprim, chloramphenicol and fosfomycin.

S1-PFGE showed that the resistance phenotype was associated with the transfer of a single plasmid (ca. 90 kb). All transconjugants were PCR positive for the NDM-1 gene. To confirm the plasmid location of the NDM-1 gene, the plasmid DNA bands from the S1-PFGE were excised from the gel and used as DNA templates for PCR. The results were positive. Replicon typing showed that the 90 kb plasmid belonged to the broad host range L/M incompatibility group (IncL/M). PCR further revealed that it encoded several antibiotic resistance genes (TEM, *armA* and *aacC2*) and the mucAB operon (UV resistance). In addition, hybridization experiments were performed by using the DIG-labeled PCR products as a probe. A single positive band was demonstrated in all the transconjugants.

### Lethal effect of UV irradiation

The mean (± standard deviation) log_10_ reduction at inoculums of 1.7×10^3^ cells and 1.7×10^4^ cells were, respectively, as follows: J53 recipient (3.0±0.2 and 2.1±0.5), pHK01-TC transconjugant (2.9±0.1 and 1.8±0.3) and NDM-TC transconjugant (1.2±0.4 and 1.0±0.3). The results showed that UV irradiation had a greater lethal effect on J53 or pHK01-TC than the NDM-TC at both inoculums (*P* = 0.002 and 0.02 for J53 versus NDM-TC; *P* = 0.3 and 0.5 for J53 versus pHK01-TC; and *P* = 0.001 and 0.03 for pHK01-TC vs. NDM-TC). The finding indicated that the transfer plasmid was capable of conferring a UV resistance phenotype.

### Analysis of pNDM-HK

**Figure 1:**
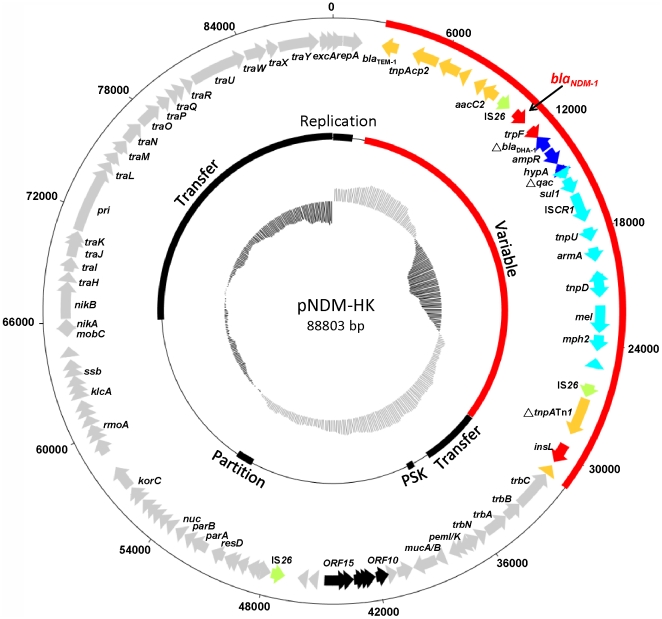
An overview of the *bla*_NDM-1_ encoding plasmid, pNDM-HK. Starting from the outside, the first circle indicates the coordinate of the complete plasmid circle and the 28.9 kb variable region is showed in red. The open reading frames (ORFs) were annotated in the second circle with arrows representing the direction of transcription. Coding sequences with and without pEL60 homologs are indicated in grey and black, respectively. All IS*26* elements are indicated in green. The variable resistant region is coded by the same color scheme as in figure 2. The third circle indicates the functional sequence blocks. The G+C plot is indicated in the inner circle (mean 51.5%), ranging from high (grey) to low (black).

The NDM-1 plasmid from the NDM-TC transconjugant was sequenced. It was a 88,803 bp plasmid with an average GC content of 51.5% and 102 open reading frames (ORFs) (Figure 1 and Table S1). Sequence alignment showed that it consisted of a plasmid backbone (approximately 55 kb) which shared extensive identity (97%) with the IncL/M plasmid, pEL60 (60145 bp, described for the plant pathogen, *Erwinia amylovora*, accession no. AY422214) and three DNA insertions (with sizes of 28.9 kb, 3522 bp and 1238 bp). The backbone part included the complete array of genes for replication, plasmid transfer, partition and stabilization, and the order of the genes and the overall organization were almost identical to those in pEL60 [12]. The backbone included the toxin-antitoxin addiction system (pemI/pemK) and the ultraviolet resistance genes (*mucAB*). The same backbone has been found in pCTX-M3 (AF550415, size 89,468 bp) and pCTX-M360 (EU938349, size 680,18 bp). The largest insertion (variable region, approximately 28.9 kb) was found between the *repA* and *trbC*. The 28.9 kb insertion included intact or truncated genes associated with resistance to β-lactams (*bla*_TEM-1_, *bla*_NDM-1_, Δ*bla*_DHA-1_), aminoglycosides (*aacC2*, *armA*), sulphonamides (*sul*1) and macrolides (*mel*, *mph2*). It also harbored the following mobile elements: IS*26*, IS*CR1*, *tnpU*, *tnpAcp2*, *tnpD*, Δ*tnpA*Tn*1* and *insL*. The other two smaller insertions (positions 41437 to 44958 and 46512 to 47749) were found downstream of ORF9 and ORF17, respectively.

The NDM-1 gene was found within the 28.9 kb variable region. Comparing to pkpANDM-1, an additional 24 bp was found in the region between *bla*_NDM-1_ and *trpF*. Blast analysis of the 28.9 kb region showed extensive homology to arrays reported in three previously sequenced plasmids [13]–[15], including pCTX-M-3 (isolated in a Polish hospital from *Citrobacter freundii* in 1996), pMUR050 (isolated from an *E. coli* isolate originating from a pig in Spain in 2002) and pKP048 (isolated in a Chinese hospital from *K. pneumoniae* in 2006).

**Figure 2:**
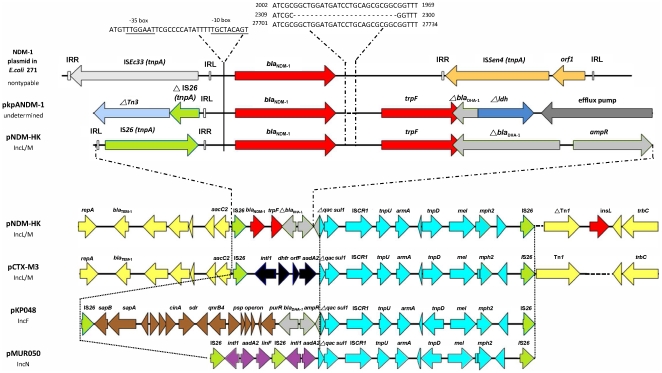
Schematic representation of the DNA sequences surrounding the *bla*_NDM-1_ genes in *E. coli* 271 NDM-1 encoding plasmid, pNDM-HK, pkpANDM-1 and comparison with the sequences in pCTX-M-3 and pKP048. (A) Comparison of the regions surrounding *bla*_NDM-1_ in the *E. coli* 271 plasmid encoding this gene, pNDM-HK, and pkpANDM-1. The 2.1 kb DNA sequence between the left inverted repeat (IRL) or right inverted repeat (IRR) and the end of phosphoribosyl anthranilate isomerase (trpF) in pkpNDM-1 and pNDM-HK were identical except for a 24 bp deletion. The sequence to the left of *trpF* up to the −35 promoter in pNDM-1HK is 100% identical to the corresponding region in the NDM-1 encoding plasmid in *E. coli* 271. The putative −35 and −10 promoter regions were indicated above and underlined. In both plasmids, this region is flanked by IS*26* and a truncated β-lactamase (*bla*_DHA-1_) gene. (B) Comparison of pNDM-HK, pCTX-M-3 and pKP048. The regions franked by IS*26* (green color) and the surrounding sequences are represented. The gaps in the alignment are shown in dotted lines. Arrows showed the direction of transcription. The same color and label are used to represent homologous genes. The lengths of the arrows are drawn in proportion to the length of the genes or open reading frames (ORFs). The homologous genes found in all three plasmids are indicated in light blue; those genes found in pNDM-HK and pCTX-M3 are in yellow; and those found in pNDM-HK and pKP048 are in blue. The genes unique to the three plasmids are labeled in red, black, brown or purple. Other abbreviations and symbols were: Δ, genes that are truncated; Tn, transposon; ldh, lactate dehydrogenase; *bla*_NDM-1_, New Delhi metallo-β-lactamase gene; *bla*_TEM-1_, class A beta-lactamase gene; *bla*_DHA-1_, class C beta-lactamase gene; *ampR*, LysR family *bla*_DHA-1_ regulator; *aacC2*, aminoglycoside acetyltransferase gene; *armA*, 16S rRNA methylase gene; *mel*, macrolide efflux protein; *mph2*, macrolide 2′-phosphotransferase gene; *intl1*, class 1 integrase; *dhfr*, dihydrofolate reductase gene; *aadA2*, aminoglycoside adenyltransferase gene; *sapB*, peptide transport system permease gene; *sapA*, peptide transport periplasmic protein, *cinA*, competence damage-inducible protein A; *sdr*, short-chain dehydrogenase/reductase gene; *qnrB4*, quinolone resistance protein; *psp operon*, transcriptional activator and phage shock proteins; *purR*, LacI family transcription regulator; and *linF*, lincosamide nucleotidyltransferase gene. The following were putative transposase: *ISCR1*, *tnpU*, *tnpAcp2*, *tnpD*, Δ *tnpA*Tn*1* and *insL*. The accession numbers were: plasmid encoding NDM-1 in E. coli 271 (HQ162469), pNDM-HK (GenBank accession HQ451074), pkpANDM-1 (FN396877), pCTX-M-3 (AF550415), pKP048 (FJ628167) and pMUR050 (AY522431).

As shown in Figure 2, the variable region in pNDM-HK (28.9 kb) and pCTX-M3 (27 kb) were both inserted between the replicon and *trb* operon. This region is mosaic with areas of high and low GC contents, suggesting that it arose from multiple genetic events. The array of ORFs in pNDM-HK and pCTX-M3 were identical except for: (1) the region between IS*26* and Δ*qac* (*bla*_NDM-1_-*trpF*- Δ*bla*_DHA-1_-*ampR* in pNDM-HK versus *intl*1-*dhfr*-*orfF*-*aadA2* in pCTX-M-3); and (2) the presence of an additional *insL* element (100% identity to IS*186* transposase in *E. coli*, accession no. GU371926) between Δ*tnpA*Tn*1* and the *trb* operon. The regions common to pNDM-HK and pCTX-M-3 in the repA-*trbC* intervening region were >99% homologous. The sequences between the two IS*26* form a complex transposon-like composite. The block from Δ*qac* to the downstream IS*26* was highly homologous (>99% identity) in pNDM-HK and the other three plasmids. On the other hand, the block spanning the upstream IS*26* and Δ*qac* was completely different in the four plasmids. In both pNDM-HK and pCTX-M-3, the *aacC2*-IS*26* junction was 169 bp in length and the sequences were 100% identical. In contrast, the junctions at IS*26*-*bla*_NDM-1_ (256 bp) and IS*26*-*intl*1 (219 bp) had different length and showed little sequence homology (42% identity). Similarly, the junctions at *ampR*-Δ*qac* (309 bp in length and included a putative *hypA* gene) and *aadA2*-Δ*qac* (163 bp) were of different lengths and the sequences had little homology. The truncated lengths of Δ*qac* were 288 bp and 348 bp in pNDM-HK and pCTX-M-3, respectively. In the regions franking the two copies of the IS*26* elements, no 8 bp target site duplication could be found.

## Discussion

We reported here the complete sequencing of the pNDM-HK plasmid. The findings highlighted the potential for this resistance determinant to spread extensively. Firstly, the NDM-1 gene was carried on a broad host range IncL/M plasmid. Thus, the plasmid might spread among members of the *Enterobacteriaceae* and Gram negative non-fermenters. This could partly explained its detection in many members of the *Enterobacteriaceae* family (*E. coli*, *K. pneumoniae*, *E, aerogenes*, *E. cloacae*, *C. freundii*, *Morganella morganii* and *Providencia* spp.) and *A. baumannii* [6], [16], [17]. The incorporation of the NDM-1 gene into the plasmid, pEL60 backbone means that bacterial pathogens in plants might serve as reservoirs in its dissemination. Foster *et al* showed that pEL60 was present in 46.9% of the *E. amylovora* strains originating from apple, pear and quince in Lebanon [12]. Plasmids (pCTX-M3 and pCTX-M36) which were derivatives of pEL60 have been associated with the widespread dissemination of CTX-M-3 in Poland and China [15], [18]. Secondly, the NDM-1 gene was surrounded by mobile genetic elements (IS*26*, IS*CR1* and transposases) which are increasingly recognized to be vehicles for dissemination of antibiotic resistance genes [19], [20]. The CTX-M type β-lactamase, 16 s rRNA methylases (*armA*) and quinolone resistance proteins (*qnr*) are some notable examples [13], [19], [21]. Thirdly, the UV resistance phenotype which was presumably mediated by the *mucAB* genes in the same plasmid might give bacteria carrying the plasmids a survival advantage in the environment [12]. This could potentially undermine effectiveness of new UVC technology for disinfection of hospital equipment and environment [22]. Lastly, as the elevated MICs to carbapenems might still be in the susceptible range, detection of fecal carriage and patient screening would be difficult [23].

Comparison of the regions surrounding NDM-1 gene shows that the mobilization of the NDM-1 gene in the different plasmids involved different genetic events in the past. In pNDM-HK, the linkage with IS*26* adds to the expanding list of β-lactamases that have been associated with this genetic element. The IS*26* element, a member of the IS*6* family, is widespread among *Enterobacteriaceae* plasmids [24]–[26]. It has been reported in the neighbor region of the CTX-M, OXA and SHV class of β-lactamases that were part of a transposon-like structure in many plasmids [26]–[29]. As in pNDM-HK, a second copy of IS*26* orientated in the same direction is often found near the *mph2* gene at a distance. In pNDM-HK, the arrays of genes between the two copies of IS*26* could be considered a putative composite transposon. As indicated by the absence of IS*26* target site duplication, it might insert by homologous recombination rather than a transposition event. The IS*26*-composite transposon containing *bla*_OXA_, *aac6'-1b-cr* and *catB4* was believed to insert itself into plasmids pEC_L8 and pEC_L46 by homologous recombination [25]. Since other mobile genetic elements (Δ*tnpA*Tn*1* and *insL*) were found, the possibility of other recombination events cannot be excluded. Among *E. coli* isolates in Germany, integration of the *bla*_CTX-M_/IS*26* transposon-like structure at the same sites in plasmids of different replicon types has been reported [30]. In addition, chromosomal integration of CTX-M-3 with two distantly located IS*26* elements has been demonstrated [31]. However, hybridization experiments showed that there was no chromosomal integration of the NDM-1 gene in the parent and transconjugant strains.

In conclusion, we reported the complete sequence of pNDM-HK encoding NDM-1 originating from an *E. coli* isolate in a patient treated in Hong Kong. The findings highlight the potential for carbapenems as well as other antibiotics to serve as driving force for the horizontal spread of the NDM-1 encoding plasmid or the IS*26* composite transposon to other bacteria. Of concern, NDM-1 is just one of the many emerging carbapenemase genes which are commonly found in multiple combinations with other β-lactamases and resistance genes in mobile genetic elements. Together, a single plasmid or composite transposon could mediate resistance to all the β-lactams and other antibiotics that are critically important for treatment of human bacterial infections. The challenge posed by NDM-1 is clearly formidable.

In Hong Kong, a territory-wide network has been set up for the coordinated surveillance of the NDM-1 and other carbapenemase genes. Since the international spread of NDM-1 has been strongly associated with medical tourism [6], many hospitals in Hong Kong have introduced a policy for active surveillance of colonization by carbapenem-resistant *Enterobacteriaceae* (CRE) on admission for patients with any history of surgery or inpatient treatment in an overseas hospital. All patients who were found to have CRE colonization would be placed under contact isolation in single rooms. Hopefully, this strategy will prevent or at least slow down the importation of NDM-1 and other transmissible carbapenemase genes into our healthcare system.

## Materials and Methods

### Microbiology methods

Bacteria were identified by conventional biochemical test and the VITEK GNI system (bioMerieux Vitek Inc., Hazelwood, MO, USA). Susceptibility of the isolates to antibiotics was determined by the disc diffusion method and Etest (AB Biodisk, Solna, Sweden). The results were interpreted according to the 2010 Clinical and Laboratory Standards Institute recommendation [4], [32]. Quality control was undertaken in accordance with the CLSI guidelines using standard strains (ATCC 25922 and 35218).

### Conjugation, PCR and hybridization

Conjugation experiments were carried out by the filtered and solid surface methods with *E. coli* J53Az^r^ as the recipient [2], [33]. In brief, overnight cultures of the bacteria were diluted in Luria Bertani (LB) and grew to late-exponential phase. Cell density was adjusted to 1.5×10^8^ cells/ml. Donor and recipient cells were mixed at 1∶2 donor-to-recipient ratio [3]. Transconjugants were selected on LB agar plates containing sodium azide (100 µg/ml) and another antibiotic (ampicillin 8 µg/ml, amikacin 16 µg/ml, gentamicin 4 µg/ml or meropenem [0.12, 0.25, 0.5, 1 and 2 µg/ml]). The transfer frequencies were expressed as the number of transconjugants per donor cell. Plasmids were sized by the S1 nuclease-PFGE method [2]. The experimental procedure was quality control by using a fully sequenced conjugative plasmid, pHK01 as a control. In each set of experiments, absence of growth of the parent and the recipient strains in the selection plates was confirmed. Previously described primers were used for detection of *aacC2* and *bla*_TEM_ and *bla*_NDM-1_ genes [2], [8], [34].

The replicon types for *E. coli* transconjugants with NDM-1 encoding plasmids were determined by a previously described scheme [35]. The method allowed recognition of the following plasmid incompatibility groups (Inc): FIA, FIB, FIC, FIIA, HI1, HI2, I1-Iγ, L/M, N, P, W, T, A/C, K, B/O, X, Y, F. Identification of the plasmid replicons was confirmed by sequencing of the PCR products. In addition, the NDM-1 PCR products were labeled by using the DIG High Prime kit (Roche Diagnostics GmbH, Penzberg, Germany) according to the manufacturer's recommendation and used as a probe for hybridization experiments to confirm the plasmid location of the NDM-1 gene.

### Ultraviolet light resistance

The lethal effects of ultraviolet (UV) light on the transconjugant (NDM-TC) was compared to the J53 recipient strain (control) at two inoculums (10^2^ and 10^3^ cells). In addition, a J53 transconjugatnt carrying the CTX-M-14 plasmid, pHK01 (about 70 kb, complete sequence in GenBank accession HM355591), J53/pHK01-TC was included for comparison. In brief, the bacteria were grown overnight on blood agar plate. The cells were suspended to a density of 0.5 McFarland standard and dilutions were made afterwards. Twenty microliters of the cell suspension containing the intended cell numbers was spread onto pairs of MH agar plates. One set of plates were irradiated in a UV crosslinker (CL-1000, Ultra-Violet Products Limited, Cambridge, UK) at a wavelength of 254 nm at energy level of 3300 µWs/cm^2^. The other set of plates was not irradiated. All the plates were then incubated at 35°C for 24 hours. The number of colonies in the plates was counted and the lethal effect of UV calculated as log_10_ reduction. The two isolates were tested in three independent experiments and the average results were used in the calculation

### Plasmid sequencing

The Illumina Genome Analyzer IIx was used for plasmid sequencing. Plasmid pNDM-HK was extracted from the NDM-TC transconjugant by using a Qiagen Large Construct kit (Qiagen, Hong Kong). Purified plasmid DNA was fragmented by nebulization. The fragments were amplified and a library constructed as described previously [36]. Based on the qPCR-quantified concentration of the barcoded plasmid library, it was diluted to generate ∼500,000 clusters and seeded with other samples in the same Solexa sample lane. Sequencing run of 76-base pair-end reads was performed according to the manufacturer's recommendations [36].

### Bioinformatics analysis

Raw data from the Solexa sequencer was analyzed with the Illumina Off-Line Basecaller Software v1.6. A phi-X 174 control lane was included in the Solexa run for matrix, phasing, and error rate estimations as recommended by the manufacturer. The error rate of the phi-X 174 control was about 0.38% for the sequencing run. Reads containing the barcode (GCTCG) and plasmid sequence were first separated from the other reads in the same lane. We then performed quality assessment on the raw reads and filtered out reads that were of low quality, adaptor sequences and homopolymer sequences. The first 6 bases that corresponded to the barcode and T-overhang were trimmed from the 76-bp reads and duplicated reads removed. The resulting high quality non-redundant data set was used as the starting material in the de novo assembly of the plasmid.

A total of 11,724,864 high-quality reads were derived from pNDM-HK library, of which 5.3% of reads were derived from trace genomic DNA contamination showing at least 90% similarity with *E. coli* genomic DNA sequences (NCBI RefSeq accession NC_000913.2) using the BLAST algorithm [37]. The assembly of the sequencing reads was carried out using *de novo* assembler Velvet (Version 0.7.62), generating 2 contigs, which were closed by additional rounds of PCR and Sanger dideoxy-sequencing to complete the assembly. Subsequently, the plasmid sequences were annotated by the RAST Server [38] and each predicted protein was further compared against the NCBI non-redundant protein database using BLASTP. The comparison of overall plasmid sequence was conducted by WebACT and Geneious Pro (Version 5.0.1, Biomatters Limited, Auckland, New Zealand). DNAplotter (Sanger Institute) was used for construction of a schematic plasmid map.

### Nucleotide sequence

The GenBank accession number for pNDM-HK plasmid is HQ451074.

## Supporting Information

Table S1 **ORFs in pNDM-HK and their annotations.**
(DOC)
