# Supplementary material for: Complete Sequencing of pNDM-HK Encoding NDM-1 Carbapenemase from a Multidrug-Resistant Escherichia coli Strain Isolated in Hong Kong
Source: PLoS One. 2011 Mar 21;6(3):e17989. doi: 10.1371/journal.pone.0017989 (PMC3061923; doi:10.1371/journal.pone.0017989)
Supplement: Table S1 — ORFs in pNDM-HK and their annotations. (DOC) [file pone.0017989.s001.doc]

Table S1. ORFs and annotation of pNDM-HK

| Gene name | presence in pEL60 | Nucleotide position | Length (bp) | Function encoded |
| --- | --- | --- | --- | --- |
| *repC* | + | 11-241 | 462 | Replication protein, RepC |
| *repB* | + | 492-542 | 51 | Replication protein, RepB |
| *repA* | + | 539-1594 | 1056 | Replication protein, RepA |
| *bla*_TEM-1_ | - | Compl. 3458-2598 | 861 | Class A beta-lactamase TEM-1 |
| *tnpAcp2* | - | Compl. 5676-4279 | 1398 | Putative transposase |
| *orf1* | - | Compl. 6921-5764 | 1158 | Hypothetical protein |
| *orf2* | - | Compl. 7178-6951 | 228 | Hypothetical protein |
| *orf3* | - | Compl. 8417-7875 | 543 | Hypothetical protein |
| *aacC2* | - | Compl. 9290-8430 | 861 | Aminoglycoside acetyltransferase |
| IS*26tnpA* | - | 9523-10227 | 705 | Transposase of IS26 |
| *bla*_NDM-1_ | - | 10536-11348 | 813 | New Delhi metallo-β-lactamase |
| *trpF* | - | 11722-12381 | 660 | Phosphoribosylanthranilate isomerase |
| *bla*_DHA-1_ | - | Compl. 13097-12303 | 795 | Class C beta-lactamase DHA-1 truncated |
| *ampR* | - | 13208-14083 | 876 | LysR family blaDHA-1 regulator |
| *hypA* | - | Compl. 14413-14087 | 327 | Putative hydrogenase nickel incorporation protein truncated |
| *qacdelta1* | - | 14393-14680 | 288 | Quaternary ammonium compound-resistance protein truncated |
| *sul1* | - | 14674-15513 | 840 | Sulfonamide resistance protein |
| IS*CR1* | - | 15918-17459 | 1542 | Putative transposase |
| *tnpU* | - | 17795-18532 | 738 | Putative transposase |
| *armA* | - | 18858-19631 | 774 | 16S rRNA methylase |
| *orf4* | - | Compl. 20298-20113 | 186 | Hypothetical protein |
| *tnpD* | - | 20347-21531 | 1185 | Putative transposase |
| *mel* | - | 21930-23405 | 1476 | Macrolide efflux protein |
| *mph2* | - | 23461-24345 | 885 | Macrolide 2'-phosphotransferase |
| *orf5* | - | Compl. 25246-24704 | 543 | Hypothetical protein |
| IS*26tnpA* | - | 26113-26835 | 723 | Transposase of IS26 |
| *tnpA*Tn*1* | - | 26889-28946 | 2058 | transposase of Tn*1* truncated |
| *insL* | - | 29530-30648 | 1119 | Putative tranposase of IS186 |
| *orf6* | - | Compl. 31359-30850 | 510 | Hypothetical protein |
| *trbC* | + | Compl. 33494-31407 | 2088 | Conjugal transfer protein, TrbC |
| *trbB* | + | Compl. 34457-33507 | 951 | Conjugal transfer protein, TrbB |
| *trbA* | + | Compl. 35775-34468 | 1308 | Conjugal transfer protein, TrbA |
| *trbN* | + | Compl. 36170-35775 | 396 | Putative lytic transglycosylase |
| *orf7* | + | Compl. 36625-36275 | 351 | Hypothetical protein |
| *tir* | + | 36738-37391 | 654 | Transfer inhibition protein |
| *pemI* | + | 37484-37741 | 258 | Stable plasmid inheritance, antitoxin |
| *pemK* | + | 37743-38075 | 333 | Stable plasmid inheritance, toxin |
| *mucA* | + | 38420-38854 | 435 | translesion synthesis protein A |
| *mucB* | + | 38803-40107 | 1305 | translesion synthesis protein B |
| *orf8* | + | Compl. 40978-40130 | 849 | Hypothetical protein |
| *orf9* | + | Compl. 41301-40981 | 321 | Hypothetical protein |
| *orf10* | - | Compl. 42138-41446 | 693 | Hypothetical protein |
| *orf11* | - | Compl. 42362-42153 | 210 | Hypothetical protein |
| *orf12* | - | Compl. 42583-42365 | 219 | Hypothetical protein |
| *orf13* | - | Compl. 43311-42628 | 684 | Hypothetical protein |
| *orf14* | - | Compl. 43580-43308 | 273 | Hypothetical protein |
| *orf15* | - | Compl. 44872-43598 | 1275 | Hypothetical protein |
| *orf16* | + | 45390-45743 | 354 | Hypothetical protein |
| *orf17* | + | 45926-46312 | 387 | Hypothetical protein |
| IS*26tnpA* | - | Compl. 47680-46976 | 705 | Transposase of IS26 |
| *orf18* | + | Compl. 48193-47744 | 450 | Hypothetical protein, truncated by the insertion of IS26 |
| *orf19* | + | 48334-48795 | 462 | Hypothetical protein |
| *orf20* | + | 48792-49040 | 249 | Hypothetical protein |
| *orf21* | + | 49033-49620 | 588 | Hypothetical protein |
| *orf22* | + | 49617-50102 | 486 | Hypothetical protein |
| *orf23* | + | 50099-50347 | 249 | Hypothetical protein |
| *resD* | + | 50366-51106 | 741 | Resolvase |
| *parA* | + | 51347-52321 | 975 | plasmid partition protein A |
| *parB* | + | 52324-52767 | 444 | plasmid partition protein B |
| *nuc* | + | 52777-53340 | 564 | Endonuclease |
| *orf24* | + | 53458-53964 | 507 | Hypothetical protein |
| *orf25* | + | 53957-54436 | 480 | Hypothetical protein |
| *orf26* | + | 54465-54875 | 411 | Hypothetical protein |
| *orf27* | + | 54993-55256 | 264 | Hypothetical protein |
| *orf28* | + | 55278-55640 | 363 | Hypothetical protein |
| *orf29* | + | 55762-56211 | 450 | Hypothetical protein |
| *korC* | + | 56256-56522 | 267 | Putative transcriptional repressor protein |
| *orf30* | + | 56586-57869 | 1284 | Hypothetical protein |
| *orf31* | + | 58729-59589 | 861 | Hypothetical protein |
| *orf32* | + | 59779-60117 | 339 | Hypothetical protein |
| *rmoA* | + | 60215-60445 | 231 | Regulatory protein |
| *orf33* | + | 60743-60979 | 237 | Hypothetical protein |
| *orf34* | + | 61064-61474 | 411 | Hypothetical protein |
| *orf35* | + | 61536-61841 | 306 | Hypothetical protein |
| *klcA* | + | 62042-62482 | 441 | Anti-restriction protein |
| *orf36* | + | 62526-62810 | 285 | Hypothetical protein |
| *orf37* | + | 62964-63184 | 221 | Hypothetical protein |
| *ssb* | + | 63245-63691 | 447 | Single-stranded binding protein |
| *orf38* | + | 63750-64061 | 312 | Hypothetical protein |
| *orf39* | + | 64194-64730 | 537 | Hypothetical protein |
| *mobC* | + | Compl. 65626-65231 | 396 | Mobility protein C |
| *nikA* | + | 65871-66188 | 318 | Mobility protein B |
| *nikB* | + | 66175-68154 | 1980 | Putative nickase |
| *traH* | + | 68168-68668 | 501 | Conjugative transfer protein |
| *traI* | + | 68665-69444 | 780 | Conjugative transfer protein |
| *traJ* | + | 69455-70618 | 1164 | Conjugative transfer protein |
| *traK* | + | 70608-70868 | 261 | Conjugative transfer protein |
| *pri* | + | 70893-74420 | 3528 | DNA primase |
| *traL* | + | 74386-74898 | 513 | Conjugative transfer protein |
| *orf40* | + | 74849-75514 | 666 | Hypothetical protein |
| *traM* | + | 75492-76274 | 783 | Conjugative transfer protein |
| *traN* | + | 76283-77434 | 1152 | Conjugative transfer protein |
| *traO* | + | 77446-78795 | 1350 | Conjugative transfer protein |
| *traP* | + | 78801-79511 | 711 | Conjugative transfer protein |
| *traQ* | + | 79535-80065 | 531 | Conjugative transfer protein |
| *traR* | + | 80082-80471 | 390 | Conjugative transfer protein |
| *orf41* | + | 80490-81011 | 522 | Hypothetical protein |
| *traU* | + | 81008-84058 | 3051 | Conjugative transfer protein |
| *traW* | + | 84055-85263 | 1209 | Conjugative transfer protein |
| *traX* | + | 85260-85910 | 651 | Conjugative transfer protein |
| *traY* | + | 85903-88083 | 2181 | Conjugative transfer protein |
| *excA* | + | 88086-88739 | 654 | Exclusion-determining protein |

pEL60 (Accession number AY422214)
